# Supplementary material for: Hot spot prediction in protein-protein interactions by an ensemble system
Source: BMC Syst Biol. 2018 Dec 31;12(Suppl 9):132. doi: 10.1186/s12918-018-0665-8 (PMC6311905; doi:10.1186/s12918-018-0665-8)
Supplement: Supplementary file 2 — All relevant MATLAB code. A simple Matlab implement of our predictor. (ZIP 19,742 kb) [file 12918_2018_665_MOESM2_ESM.zip › Additional file 2/Read ASA/ASA website/SKEMPI/1EMVB.htm]

 NetSurfP - Protein Surface Accessibility and Secondary Structure Predictions

  

|  |  |  |
| --- | --- | --- |
|  |  | NetSurfP - Protein Surface Accessibility and Secondary Structure Predictions  Technical University of Denmark |

  


---

```
# For publication of results, please cite:
# A generic method for assignment of reliability scores applied to solvent accessibility predictions.
# Bent Petersen, Thomas Nordahl Petersen, Pernille Andersen, Morten Nielsen and Claus Lundegaard
# BMC Structural Biology 2009, 9:51 doi:10.1186/1472-6807-9-51
#
# Column 1: Class assignment - B for buried or E for Exposed - Threshold: 25% exposure, but not based on RSA
# Column 2: Amino acid
# Column 3: Sequence name
# Column 4: Amino acid number
# Column 5: Relative Surface Accessibility - RSA
# Column 6: Absolute Surface Accessibility
# Column 7: Z-fit score for RSA prediction
# Column 8: Probability for Alpha-Helix
# Column 9: Probability for Beta-strand
# Column 10: Probability for Coil
E M  1EMVB                  1    0.692 138.549  -2.591   0.003   0.003   0.994
E E  1EMVB                  2    0.669 116.839   0.192   0.018   0.019   0.964
E S  1EMVB                  3    0.441  51.638  -1.553   0.018   0.019   0.964
E K  1EMVB                  4    0.435  89.541  -0.593   0.053   0.043   0.903
E R  1EMVB                  5    0.433  99.180  -0.661   0.053   0.043   0.903
E N  1EMVB                  6    0.517  75.660  -0.867   0.018   0.047   0.935
E K  1EMVB                  7    0.540 111.099  -0.348   0.018   0.047   0.935
E P  1EMVB                  8    0.398  56.490  -1.187   0.018   0.047   0.935
B G  1EMVB                  9    0.253  19.927  -2.194   0.020   0.205   0.775
E K  1EMVB                 10    0.376  77.240  -0.093   0.004   0.514   0.481
B A  1EMVB                 11    0.131  14.414  -1.395   0.023   0.655   0.322
E T  1EMVB                 12    0.337  46.784  -0.414   0.022   0.552   0.426
B G  1EMVB                 13    0.279  21.942  -2.228   0.021   0.451   0.528
E K  1EMVB                 14    0.520 107.046   0.355   0.022   0.359   0.619
E G  1EMVB                 15    0.426  33.518  -1.603   0.020   0.205   0.775
E K  1EMVB                 16    0.439  90.220   0.838   0.022   0.552   0.426
E P  1EMVB                 17    0.576  81.777   0.321   0.023   0.655   0.322
B V  1EMVB                 18    0.117  17.968  -0.153   0.074   0.484   0.442
E G  1EMVB                 19    0.347  27.309  -1.116   0.135   0.317   0.548
E D  1EMVB                 20    0.347  50.075  -1.375   0.216   0.235   0.548
E K  1EMVB                 21    0.399  82.033   0.108   0.231   0.330   0.439
B W  1EMVB                 22    0.139  33.478   0.121   0.453   0.248   0.299
B L  1EMVB                 23    0.174  31.786   0.559   0.605   0.105   0.290
E D  1EMVB                 24    0.397  57.208  -1.044   0.561   0.047   0.393
B D  1EMVB                 25    0.254  36.616  -1.898   0.522   0.016   0.462
B A  1EMVB                 26    0.244  26.878  -1.252   0.339   0.016   0.645
B G  1EMVB                 27    0.275  21.627  -1.699   0.257   0.016   0.727
E K  1EMVB                 28    0.557 114.595   0.024   0.115   0.016   0.868
E D  1EMVB                 29    0.483  69.658  -2.534   0.058   0.017   0.925
E S  1EMVB                 30    0.381  44.653  -1.384   0.053   0.043   0.903
B G  1EMVB                 31    0.233  18.313  -2.095   0.018   0.047   0.935
B A  1EMVB                 32    0.123  13.588  -0.432   0.056   0.142   0.802
B P  1EMVB                 33    0.217  30.736  -0.872   0.118   0.150   0.732
B I  1EMVB                 34    0.105  19.499   0.971   0.191   0.086   0.723
B P  1EMVB                 35    0.068   9.692   0.053   0.717   0.014   0.269
E D  1EMVB                 36    0.347  50.075   0.366   0.923   0.002   0.076
E R  1EMVB                 37    0.346  79.234   0.743   0.923   0.002   0.076
B I  1EMVB                 38    0.030   5.476   0.314   0.923   0.002   0.076
B A  1EMVB                 39    0.075   8.254  -0.101   0.923   0.002   0.076
E D  1EMVB                 40    0.409  58.879  -0.004   0.858   0.002   0.139
E K  1EMVB                 41    0.326  67.161   1.058   0.782   0.003   0.216
B L  1EMVB                 42    0.073  13.348  -0.643   0.717   0.014   0.269
E R  1EMVB                 43    0.379  86.699   0.384   0.430   0.016   0.555
E D  1EMVB                 44    0.488  70.306  -0.977   0.257   0.016   0.727
E K  1EMVB                 45    0.413  85.036   0.683   0.115   0.016   0.868
E E  1EMVB                 46    0.534  93.290   0.485   0.113   0.043   0.844
B F  1EMVB                 47    0.125  24.987  -0.091   0.053   0.043   0.903
E K  1EMVB                 48    0.531 109.268  -0.291   0.018   0.019   0.964
E S  1EMVB                 49    0.352  41.266  -0.481   0.018   0.019   0.964
B F  1EMVB                 50    0.103  20.612   0.581   0.321   0.003   0.675
E D  1EMVB                 51    0.515  74.240   0.353   0.694   0.003   0.303
E D  1EMVB                 52    0.391  56.415   0.703   0.782   0.003   0.216
B F  1EMVB                 53    0.039   7.928   0.043   0.923   0.002   0.076
B R  1EMVB                 54    0.096  22.030   0.785   0.970   0.001   0.030
E K  1EMVB                 55    0.450  92.462   0.973   0.970   0.001   0.030
B A  1EMVB                 56    0.213  23.462   0.714   0.970   0.001   0.030
B V  1EMVB                 57    0.035   5.410   0.551   0.970   0.001   0.030
B W  1EMVB                 58    0.063  15.200   0.402   0.970   0.001   0.030
E E  1EMVB                 59    0.338  58.961   1.623   0.923   0.002   0.076
B E  1EMVB                 60    0.141  24.650   0.202   0.938   0.007   0.055
B V  1EMVB                 61    0.042   6.394   0.887   0.802   0.014   0.185
B S  1EMVB                 62    0.201  23.534  -0.457   0.430   0.016   0.555
E K  1EMVB                 63    0.657 135.186   0.328   0.109   0.005   0.886
B D  1EMVB                 64    0.231  33.273   0.216   0.053   0.005   0.942
E P  1EMVB                 65    0.408  57.924  -0.196   0.600   0.003   0.397
E E  1EMVB                 66    0.566  98.863   0.506   0.802   0.014   0.185
B L  1EMVB                 67    0.085  15.490   0.630   0.802   0.014   0.185
B S  1EMVB                 68    0.093  10.911  -0.117   0.802   0.014   0.185
E K  1EMVB                 69    0.602 123.893   0.846   0.717   0.014   0.269
E N  1EMVB                 70    0.309  45.311   0.157   0.522   0.016   0.462
B L  1EMVB                 71    0.060  11.078   0.435   0.339   0.016   0.645
E N  1EMVB                 72    0.367  53.729   0.705   0.115   0.016   0.868
E P  1EMVB                 73    0.639  90.688   0.213   0.430   0.016   0.555
E S  1EMVB                 74    0.453  53.056  -0.835   0.406   0.004   0.590
B N  1EMVB                 75    0.148  21.667  -1.271   0.406   0.004   0.590
E K  1EMVB                 76    0.259  53.338   0.501   0.802   0.014   0.185
E S  1EMVB                 77    0.520  60.979   0.557   0.802   0.014   0.185
B S  1EMVB                 78    0.191  22.420   0.891   0.802   0.014   0.185
B V  1EMVB                 79    0.073  11.205   0.146   0.802   0.014   0.185
E S  1EMVB                 80    0.507  59.479   0.553   0.717   0.014   0.269
E K  1EMVB                 81    0.612 125.827   0.203   0.339   0.016   0.645
E G  1EMVB                 82    0.389  30.591  -1.815   0.115   0.016   0.868
E Y  1EMVB                 83    0.305  65.178  -0.795   0.018   0.019   0.964
B S  1EMVB                 84    0.194  22.795  -0.691   0.018   0.047   0.935
B P  1EMVB                 85    0.257  36.412  -1.455   0.018   0.047   0.935
E F  1EMVB                 86    0.373  74.861  -0.518   0.018   0.088   0.893
B T  1EMVB                 87    0.136  18.835  -0.468   0.018   0.088   0.893
E P  1EMVB                 88    0.285  40.456   0.788   0.018   0.088   0.893
E K  1EMVB                 89    0.407  83.802  -0.539   0.053   0.043   0.903
E N  1EMVB                 90    0.575  84.239  -1.203   0.113   0.087   0.800
E Q  1EMVB                 91    0.584 104.231  -1.449   0.118   0.150   0.732
E Q  1EMVB                 92    0.435  77.762  -0.258   0.125   0.227   0.648
B V  1EMVB                 93    0.228  35.090  -1.288   0.064   0.216   0.721
E G  1EMVB                 94    0.468  36.800  -1.258   0.018   0.088   0.893
E G  1EMVB                 95    0.508  39.956  -0.856   0.018   0.088   0.893
E R  1EMVB                 96    0.412  94.233  -0.006   0.005   0.262   0.733
E K  1EMVB                 97    0.400  82.342   0.210   0.004   0.514   0.481
E V  1EMVB                 98    0.429  66.014   1.674   0.002   0.816   0.182
B Y  1EMVB                 99    0.075  16.006   0.485   0.001   0.900   0.099
B E  1EMVB                100    0.198  34.538   0.533   0.001   0.900   0.099
B L  1EMVB                101    0.038   7.013   0.452   0.002   0.816   0.182
B H  1EMVB                102    0.199  36.162   1.032   0.003   0.718   0.279
B H  1EMVB                103    0.121  22.046  -0.046   0.023   0.655   0.322
E D  1EMVB                104    0.335  48.259   0.418   0.022   0.552   0.426
E K  1EMVB                105    0.337  69.321   0.468   0.021   0.451   0.528
E P  1EMVB                106    0.407  57.810   0.534   0.021   0.451   0.528
B I  1EMVB                107    0.087  16.095   0.294   0.021   0.451   0.528
E S  1EMVB                108    0.491  57.580   0.200   0.021   0.279   0.699
E Q  1EMVB                109    0.556  99.302  -0.468   0.019   0.141   0.840
E G  1EMVB                110    0.401  31.551  -1.451   0.018   0.047   0.935
B G  1EMVB                111    0.166  13.072  -1.992   0.018   0.047   0.935
B E  1EMVB                112    0.191  33.333  -0.762   0.021   0.279   0.699
B V  1EMVB                113    0.080  12.250  -0.331   0.022   0.552   0.426
B Y  1EMVB                114    0.108  23.058   1.234   0.022   0.552   0.426
B D  1EMVB                115    0.181  26.039  -0.097   0.021   0.451   0.528
B M  1EMVB                116    0.060  12.006   0.347   0.020   0.205   0.775
E D  1EMVB                117    0.272  39.181  -0.540   0.052   0.084   0.864
B N  1EMVB                118    0.172  25.225  -0.531   0.056   0.142   0.802
B I  1EMVB                119    0.037   6.826   0.390   0.074   0.484   0.442
B R  1EMVB                120    0.166  37.900   0.896   0.021   0.756   0.223
B V  1EMVB                121    0.043   6.655   0.122   0.021   0.756   0.223
B T  1EMVB                122    0.118  16.353   0.359   0.022   0.552   0.426
B T  1EMVB                123    0.098  13.551   0.601   0.021   0.279   0.699
B P  1EMVB                124    0.141  19.994  -0.493   0.056   0.142   0.802
E K  1EMVB                125    0.393  80.881  -0.051   0.113   0.087   0.800
E R  1EMVB                126    0.392  89.860  -0.613   0.113   0.087   0.800
B H  1EMVB                127    0.274  49.913  -0.885   0.199   0.152   0.649
E I  1EMVB                128    0.338  62.493  -1.211   0.118   0.150   0.732
E D  1EMVB                129    0.580  83.621  -0.807   0.118   0.150   0.732
E I  1EMVB                130    0.381  70.541  -0.454   0.113   0.087   0.800
E H  1EMVB                131    0.664 120.709  -1.840   0.003   0.003   0.994

Explain the output.  Go back.
```

---
